# Quartz-enhanced photoacoustic spectroscopy employing pilot line manufactured custom tuning forks

**DOI:** 10.1016/j.pacs.2019.100158

**Published:** 2019-12-26

**Authors:** Huadan Zheng, Yihua Liu, Haoyang Lin, Bin Liu, Xiaohang Gu, Dongquan Li, Bincheng Huang, Yichao Wu, Linpeng Dong, Wenguo Zhu, Jieyuan Tang, Heyuan Guan, Huihui Lu, Yongchun Zhong, Junbin Fang, Yunhan Luo, Jun Zhang, Jianhui Yu, Zhe Chen, Frank K. Tittel

**Affiliations:** a Guangdong Provincial Key Laboratory of Optical Fiber Sensing and Communications, Jinan University, Guangzhou, 510632, China; b Key Laboratory of Optoelectronic Information and Sensing Technologies of Guangdong Higher Education Institutes, Department of Optoelectronic Engineering, Jinan University, Guangzhou, 510632, China; c Guangdong Provincial Engineering Technology Research Center on Visible Light Communication and the Guangzhou Municipal Key Laboratory of Engineering Technology on Visible Light Communication, Jinan University, Guangzhou, 510632, China; d School of Physics and Optoelectronic Engineering, Foshan University, Foshan, 528000, China; e Department of Electrical and Computer Engineering, University of Washington, Seattle, Washington 98195, USA; f Department of Electrical and Computer Engineering, Rice University, Houston, Texas 77005, USA

**Keywords:** Photoacoustic spectroscopy, Photoacoustic detection, Gas sensing, Quartz tuning fork

## Abstract

Pilot line manufactured custom quartz tuning forks (QTFs) with a resonance frequency of 28 kHz and a Q value of >30, 000 in a vacuum and ∼ 7500 in the air, were designed and produced for trace gas sensing based on quartz enhanced photoacoustic spectroscopy (QEPAS). The pilot line was able to produce hundreds of low-frequency custom QTFs with small frequency shift < 10 ppm, benefiting the detecting of molecules with slow vibrational-translational (V-T) relaxation rates. An Au film with a thickness of 600 nm were deposited on both sides of QTF to enhance the piezoelectric charge collection efficiency and reduce the environmental electromagnetic noise. The laser focus position and modulation depth were optimized. With an integration time of 84 s, a normalized noise equivalent absorption (NNEA) coefficient of 1.7 × 10^−8^ cm^-1^∙W∙Hz^-1/2^ was achieved which is ∼10 times higher than a commercially available QTF with a resonance frequency of 32 kHz.

## Highlights

• Pilot line manufactured custom quartz tuning forks (QTFs) with a resonance frequency of 28 kHz and a Q value of >30, 000 in a vacuum and ∼ 7500 in the air, were designed.
• The pilot line was able to produce hundreds of custom QTFs with small frequency shift < 10 ppm.
• An Au film were deposited on both sides of QTF to enhance the piezoelectric charge collection efficiency and reduce the environmental electromagnetic noise.
• The laser focus position and modulation depth were optimized to enhance the laser excitation efficiency.
• A normalized noise equivalent absorption (NNEA) coefficient of 1.7 × 10^−8^ cm^−1^ W Hz^−1/2^ was achieved.

## 1 Introduction

Laser photoacoustic spectroscopy (PAS) for trace gas detection has been widely investigated and applied in recent decades [[1], [2], [3]]. As a variation of PAS, quartz-enhanced photoacoustic spectroscopy (QEPAS) [[4], [5], [6], [7], [8], [9]], is a particularly sensitive gas detection technique capable of trace gas detection at the parts-per-trillion (ppt) level [10]. Since the first demonstration of QEPAS in 2002 [4], gas sensors based on QEPAS have been widely used for environmental monitoring, industrial process control and clinical diagnostics [[11], [12], [13], [14], [15], [16], [17], [18]]. The significant advantage of QEPAS is to accumulate the photoacoustic energy in an extremely sharp resonant quartz tuning fork (QTF), which acts as a piezoelectric acoustic transducer instead of a conventional microphone [[19], [20], [21], [22], [23]]. The acoustic wave induced by photoacoustic effect and applied on the prong of the QTF is converted into electric signal by the piezoelectric effect of the QTF. The high resonance frequency of 32 kHz and its narrow bandwidth of ∼ 4 Hz result in a relatively high *Q* factor and good environmental acoustic noise immunity when a employing a commercial QTF.

The signal amplitude of the QEPAS is given by equation 1 [24]:

(1) $$S\propto \frac{\alpha PQ}{f_{0}}$$

where α, P, $f_{0}$ are the gas absorption coefficient, the laser power and the QTF resonance frequency, respectively. To ensure that the molecular vibration to translation (V-T) relaxation following the laser modulation frequency, a condition that the molecular relaxation time τ should be shorter than the modulation period *τ*≪1/*f* should be satisfied. Otherwise this could lead to a signal amplitude reduction or a phase shift of the photoacoustic signal when using QEPAS to detect molecules with a slow V-T relaxation [25,26]. For example, in the case of a dry CO_2_-N_2_ gas mixture, the relaxation time reached a value of >100 μs, leading to a signal reduction of 60 % if a 32,768 Hz commercial QTF was employed as the photoacoustic transducer [25]. Until 2013, custom QTFs with prong spacings of up to 1.5 mm and low resonance frequencies down to 2.8 kHz were investigated [27,28]. In 2015, QTF with a resonance frequency of 30.72 kHz was used as photoacoustic transducer to enhance the QEPAS signal amplitude [29]. Most recently, custom QTFs with optimized geometries for a QEPAS spectrophone was demonstrated [30].

Although custom QTFs with different frequencies were employed in QEPAS most recently, however due to the fineness of the manufacture technique the designed QTF has different frequency shift. To the best of our knowledge, it is the first time that pilot line manufactured custom tuning forks was developed. In this manuscript, high performance custom quartz tuning fork (QTF) was designed for trace gas sensing based on quartz enhanced photoacoustic spectroscopy (QEPAS). The developed custom QTFs have the resonance frequencies down to 28 kHz while remaining the nearly the same size as the commercial QTF with the resonance frequency of 32 kHz. Unlike traditional custom QTF, the developed custom QTF showed the uniform resonance frequency with a shift < 10 ppm manufactured by a pilot line. An Au film with a thickness of 600 nm are deposited on both sides of QTF to enhance the piezoelectric charge collection efficiency and reduce the environmental electromagnetic noise. The QEPAS sensor performance based on the custom QTF was evaluated by detecting the H_2_O in the ambient air. Laser focus position effect to improve the excitation efficiency in the QEPAS was investigated both theatrically and experimentally. Allan deviation confirms a good long-term stability of the QEPAS sensor.

## 2 Sensor design

**Fig. 1:**
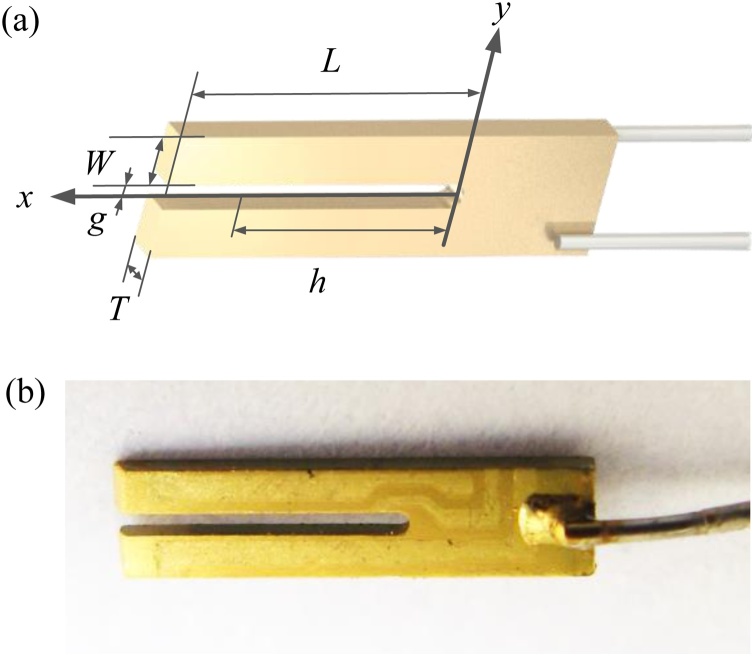
a) Diagram of the QTF dimension; (b) Photograph of the QTF taken with an optical microscope.

**Fig. 2:**
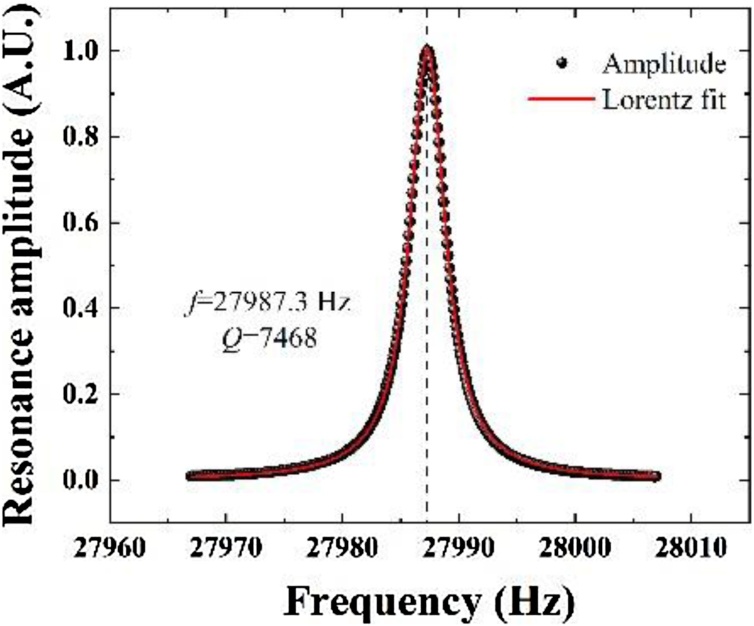
Resonance curve of the custom 28 kHz QTF. Lorentz function is used to fit the data and calculate the frequency and *Q* factor.

**Fig. 3:**
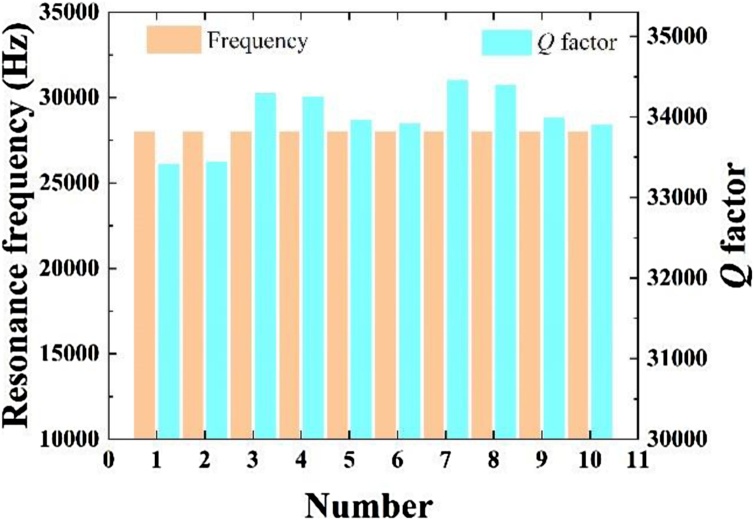
Variation of resonance frequency and Q factor of ten custom QTFs.

The custom QTF was etched using microelectronic clean room techniques from 350 μm thick Z-cut quartz wafers with the QTF prongs being oriented along the y-axis, see Fig. 1(a). The QTF model was generated by chemical etching in a hydrogen fluoride solution and then micro electrodes were protected using shadow masks. Au films with a thickness of 600 nm are deposited on both sides of the prongs of the tuning fork using vacuum coating technology to enhance the piezoelectric charge collection efficiency, see Fig.1 (b). According to the analytic solution for the flexural vibration resonance given by Ref. 31, the resonance frequency *f*_0_ of QTF can be specified as:

(2) $$f_{0}=\frac{\pi W}{8\sqrt{12}l^{2}}\sqrt{\frac{E}{\rho }}\nu_{0}^{2}$$

where *W*, *g*, *T* and *l* were defined in Fig.1(a). The Young modulus *E* and density *ρ* of quartz were 0.72 × 10^11^ N/m^2^ and 2650 Kg/m^3^ respectively. *υ*_0_ was 1.194 for the fundamental resonance [31]. The resonance frequency *f* and quality factor *Q* can be obtained from a Lorentz fit of the QTF resonance curve measured by an electrical circuit. The obtained QTF resonance curve in the air with the pressure of ∼ 747 Torr was plotted in Fig. 2. The corresponding QTF geometrical parameters and electrical parameters were shown in Table 1. The resonance frequency and *Q* factor was measured as 27987 Hz and 7463 respectively. The resistance *R* obtained by an equivalent *RLC* circuit was 220.51 kΩ. The *Q* factor can be enhanced in a lower pressure. The resonance frequencies and *Q* factor of ten custom QTFs encapsulated in a quasi-vacuum were measured to evaluate the stability of the pilot line manufacturing. The parameters of ten subjects from 100 custom QTFs manufactured by the pilot line were shown in Table 2. The mean value of the resonance frequency was calculated as 27.99 kHz which is approximately equal to the theoretical value of 28 kHz. The standard deviation of resonance frequency was 0.26 Hz corresponding to a frequency shift of 9.28 ppm. The minor frequency shift can be attributed to the manufacture technology, electrical circuit and error of Lorentz fitting. Fig. 3 shows the variations of resonance frequency and *Q* factor value of the ten custom QTFs. The obtained mean *Q* factor values was as high as ∼34, 000 in a quasi-vacuum. The slight fluctuation in Q factors comes from the gold film which was deposited by vacuum evaporation. The heterogeneity of the gold film resulted in the fluctuations in Q factor. Improvement can be made by using sputtering technology to form a uniform gold film on the QTF surface.

**Table 1:** QTF geometrical parameters and electrical parameters.

Geometrical Parameters				Electrical Parameters		
*W*(mm)	*g*(mm)	*L*(mm)	*T*(mm)	*f*(Hz)	*Q*	*R*(kΩ)
0.4	0.2	3.3	0.35	27987	7463	220.51

**Table 2:** Parameters of ten custom QTFs manufactured by the pilot line.

*f*(Hz)	*Q*	*R*(kΩ)
27997.3	33408	43.69
27997.3	33435	43.73
27997.3	34287	42.4
27997.3	34240	42.24
27997.4	33957	45.15
27997.4	33914	45.05
27997.9	34451	43.5
27997.9	34387	43.54
27997.2	33982	40.82
27997.2	33892	40.91

## 3 Experimental setup

**Fig. 4:**
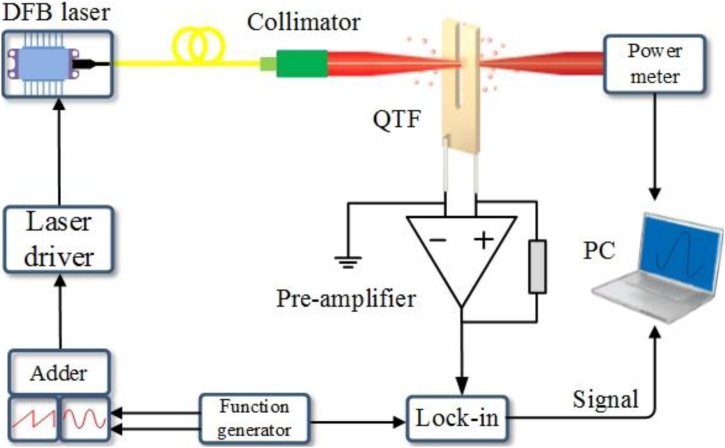
Schematic diagram of the QEPAS experimental setup. The double channel function generator produces a ramp signal with a frequency of 10 mHz and a sine signal with the frequency of 14 kHz to tune and modulate the DFB laser, respectively. PM: power meter, PC: personal computer.

The schematic diagram of the experimental setup is depicted in Fig. 4. A custom QTF with a frequency of ∼27.99 kHz and *Q* factor value of ∼33,900 in a quasi-vacuum was employed as the acoustic-electric transducer. The resonance frequency and *Q* factor value shifted to 27.98 kHz and ∼7500 in the ambient air, due to the air damping. The custom QTF has a geometry with a prong length of 3.3 mm and is ∼10 % times smaller in size with respect to a commercial QTF. A pigtailed distributed feedback (DFB) laser emitting at 1392 nm was employed to generate the photoacoustic signal. The coarse and fine tuning of laser emission wavelength can be realized by changing the temperature of laser diode and injection current of the laser diode. The 2*f* wavelength modulation technique was applied to the QEPAS to increase its signal-to-noise ratio (SNR). The laser current was sinusoidally modulated at *f*/2 of ∼14 kHz by a dual-channel function generator (Tektronix AFG 3102), where *f* is the fundamental resonance frequency of the QTF. The piezoelectric signal generated by the QTF was pre-amplified by a custom transimpedance amplifier with a feedback resistance of 10 MΩ and then fed to a lock-in amplifier (Stanford SR830 DSP) to demodulate the signal in the second harmonic mode. The time constant and filter slope of the lock-in amplifier in this experiment was set to 1 s and 12 dB/Oct respectively. A personal computer (PC) equipped with a data acquisition (DAQ) card was used to record and analyze the experimental data. The QTF was placed in an enclosure filling with air samples. The H_2_O concentration of 1.3 % was verified by means of direct absorption spectroscopy as our previous publications [32]. The experiment was conducted at atmospheric pressure of ∼ 747 Torr and room temperature of ∼25 °C. A H_2_O absorption line falling at 7194.8 cm^−1^ with an intensity of 3.07 × 10^-21^ cm/mol was selected as the target absorption line.

## 4 Experimental results

### 4.1 Laser focusing position effects and modulation depth optimization

**Fig. 5:**
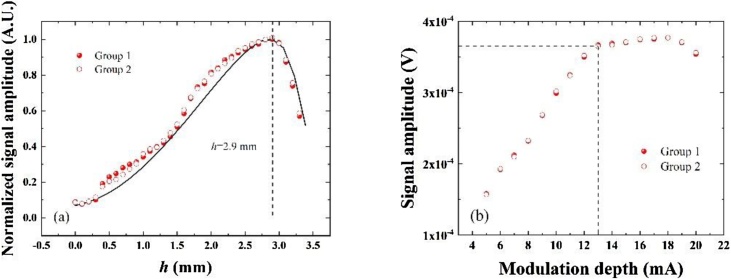
(a) Optimization of the laser focus position. The red dots, red circles and black solid lines represent the experimental group 1, experimental group 2 and numerical results, respectively. (b) Optimization of the laser modulation depth in the QEPAS based on a 28 kHz custom QTF. The red dots and circles represent experimental group 1 and experimental group 2, respectively.

In the construction of QEPAS spectrophone, there are focusing position effects along the QTF prong that must be considered when the laser focus position varies along the QTF prong [19]. The impact of laser focus position with respect to QTF on signal amplitude was investigated. The laser beam was focused between the QTF prongs and centered on the *x* axis as shown in Fig. 1(a). The value of *h* denotes the distance between the laser focus position and the junction of the QTF prongs. The position of the optical fiber focuser was adjusted by an XYZ linear translation stage with a resolution of 0.01 mm. The normalized QEPAS signal amplitudes obtained by experiment and theoretical analysis as the function of *h* are plotted in Fig. 5(a). A position to obtain the maximum signal amplitude was *h* = 2.9 mm.

A mathematical model including the generation of sound wave, motion of the QTF prong and converting the oscillation of QTF prongs to piezoelectric signals was developed to evaluate the QEPAS sensor based on the custom QTF. The sound wave pressure *P* in space and motion of the QTF prong satisfies the Eqs. (3) and (4) [33]:

(3) $$\frac{\partial^{2}P}{\partial t^{2}}-c^{2}\Delta P=S$$

(4) $$\frac{EI}{\rho A}\frac{\partial^{4}u}{\partial y^{4}}+2\beta \frac{du}{dt}+\frac{\partial^{2}u}{\partial t^{2}}=\frac{1}{\rho A}f(y,\;t)$$

where *t* is time, *c* is sound speed, and *S* is the acoustic source term. *E*, *I*, ρ, *A*, and *u*(*y*, *t*) are the Young’s modulus of quartz, the second moment of area, the density of quartz, the cross-sectional area, and the displacement at time *t* of a point at position *y* respectively. As a result, the optimum laser focus position obtained by the numerical method is well consistent with the experimental results, as shown in Fig. 5(a).

Since a 2*f* wavelength modulation technique was applied to the QEPAS, the optimum laser modulation depth must be characterized for a custom QTF. The laser temperature was fixed at 17.5 °C and the laser injection current was varied from 40 mA to 60 mA in steps of 0.1 mA to cover the selected H_2_O absorption line. The modulation depth was changed from 5 mA to 20 mA to obtain the maximum 2*f* QEPAS signal amplitude. The experimental result in Fig. 5(b) shows that the signal amplitudes increase monotonically with the laser modulation depth from 5 mA to 18 mA, whereas when the modulation depth was larger than 13 mA the QEPAS signal amplitudes increase less than 3 %. The 2*f* signal amplitudes then start to decrease when the modulation depth is larger than 18 mA, indicating that the optimum modulation depth was 13 mA. Two groups of experimental results show a consistent result.

### 4.2 QEPAS signal evaluation

**Fig. 6:**
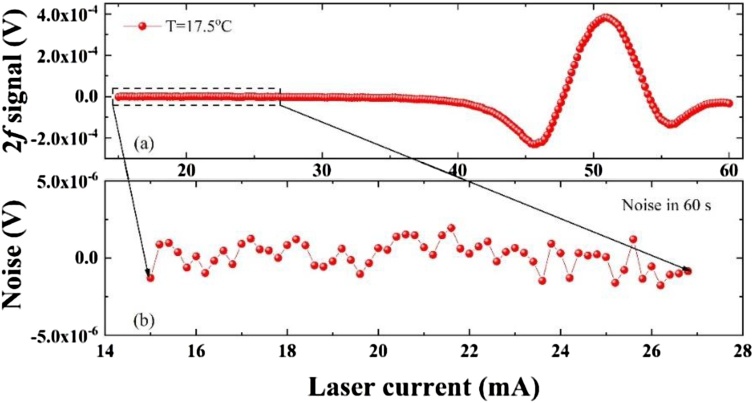
(a) QEPAS 2*f* signal of H_2_O detected in laboratory ambient air; (b) Noise obtained at the laser wavelength beyond the H_2_O absorption line.

The performance of the QEPAS sensor based on a custom QTF was evaluated by the detection of H_2_O in ambient air in a constant environmental temperature and humidity laboratory. With the laser injection current tuning from 15 mA to 60 mA, the obtained QEPAS 2*f* signal and associated noise were plotted in Fig. 6. The 2*f* signal and noise were obtained for the condition of optimum laser focus position and modulation depth of 13 mA. The signal peak of the QEPAS 2*f* signal was 3.82 × 10^−4^ V. A 1σ noise of 7.8 × 10^-7^ V was calculated from the standard deviation of the QEPAS signal when the laser emission wavelength was far from the H_2_O absorption line. As a result, the detection signal to noise ratio was calculated to be ∼490. This low 1σ noise can be attributed to the good anti-electromagnetic disturbance ability of the Au film deposited on the QTF surface.

### 4.3 Sensor long-term stability

**Fig. 7:**
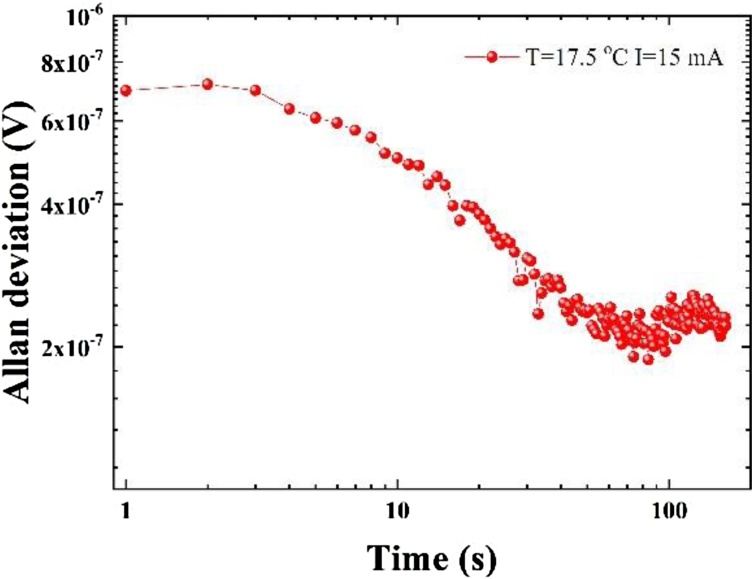
The obtained Allan deviation when the laser emission wavelength was tuned away from the H_2_O absorption.

The Allan deviation is the square root of Allan variance, which is also known as two-sample variance, is a measure of frequency stability of devices and instruments. The Allan deviation analysis allows the determination of how long optical sensor signals can be averaged to increase the detection sensitivity, and before noise sources like laser instability, temperature, and mechanical drifts, as well as when moving fringes begin to dominate [45]. To assess the long-term stability, the laser emission wavelength was tuned away from the H_2_O absorption by adjusting the laser to T = 17.5 °C and I = 15 mA, respectively. The lock-in amplifier continuously recorded the data from the QEPAS sensor with an integration time of 1 s and slope of 12 dB/octave. An Allan deviation analysis was carried out as depicted in Fig. 7. The white noise remains the dominant noise source until 84 s. After that, the instrumental drift started to dominate. With an integration time of 84 s, a SNR of 2042 was achieved, corresponding to a NNEA of 1.7 × 10^−8^ cm^-1^∙W∙Hz^-1/2^.

## 5 Discussions

For a side by side comparison, the results obtained by several QTFs were demonstrated in Table 3. According to the Eq. (1), the signal amplitude of the QEPAS sensor is proportional to the gas absorption coefficient *α*, the laser power *P*, and inversely proportional to the QTF resonance frequency *f*_0_, respectively. The gas absorption coefficient *α* was determined by the laser wavelength which resonates with the rotational and vibrational energy levels of the molecules. Laser sources with larger power, targeting a strong absorption line, will result in better a detection limit. Resonant enhancement method, including overtone resonance [28,41], single-tube on-beam configuration [28,41], on-beam configuration [39,40] and off-beam configuration [42] offers an enhancement factor of dozens of times or more. With the purpose of evaluating the performance of the proposed QTF, no resonant enhancement was employed in the QEPAS sensor. After checking the laser diodes in stock, a laser diode with the wavelength of 1392 nm and the power of 5 mW was used to target a H_2_O absorption line of 3.07 × 10^−21^ *cm/mol*. Even a lower power and weaker absorption, the detection limit of this this work was comparable to the commercial QTFs and other custom QTFs. For the improvement, an on-beam and single-tube on-beam configuration can be used to improve the detection limit by 30 times [43] and >100 times [21], respectively.

**Table 3:** Side by side comparison of QEPAS sensor based on different QTFs for H_2_O detection. Overtone: overtone enhancement; sing-tube: single-tube on-beam configuration; *f*: QTF resonance frequency; *λ*: laser wavelength; *P*: laser power; *α*: absorption line intensity; *D*: detection limit; *Ref*: reference.

Resonant Enhancement	f_0_ (kHz)	λ (nm)	P (mW)	α (cm/mol)	D (ppm)	Ref
Overtone + Single-tube	25.4	7713	108	1.70 × 10^−22^	4.59	[28]
Overtone + Single-tube	17.7	1370	23	8.06 × 10^−22^	4.3	[41]
On-beam configuration	30.7	1395	30	1.17 × 10^−20^	4.3	[40]
On-beam configuration	32.7	1395	\	\	5.73	[39]
Off-beam configuration	32.7	1396	8	1.17 × 10^−20^	9.27	[42]
None	28	1392	5	3.07 × 10^−21^	6.3	This work

## 6 Conclusions

From 2014, a series of research on QEPAS by using of different custom QTF were demonstrated [[26], [27], [28],[34], [35], [36], [37]]. Detailed experimental and theoretical analysis on the influences of the custom QTFs including the quality factor Q, the resonance frequency, the fork stiffness, the spring constant, and the electrical resistance were reported. However, the uniform in frequency of the custom QTF is never reported. Although the frequency shift can be compensated by a lock-in amplifier in the laboratory. For an efficient harmonic demodulation by using of a cost-effective lock-in module, the resonance frequencies of the QTF should be uniform with a small discrepancy in a given frequency range. The proposed custom QTF can also benefit the novel QEPAS spectrophone such as multi-quartz-enhanced photoacoustic spectroscopy [46] and the optical chopper based on QTFs [47], where the uniform resonance frequency of multi QTFs was required. In this work, we demonstrated the realization of a QEPAS gas sensing using pilot line manufactured custom QTFs for the first time. Ten custom QTFs as mechanical oscillators were characterized by the resonance frequency of ∼28 kHz with a shift of less than 10 ppm. The *Q* factor obtained by such custom QTFs were ∼34, 000 in a quasi-vacuum and ∼ 7500 in the air. A small gap of ∼200 μm benefited a higher acoustic wave pressure on the QTF prongs in the QEPAS. The electrodes, made of an Au film with a thickness of 600 nm, are deposited on both sides of the prongs of the tuning fork to increase the collection efficiency of the piezoelectric charge. The optimum laser focus position was found to be 0.4 mm away from the QTF opening, which is consistent with the theoretical value. The laser modulation depth was optimized to increase the QEPAS signal amplitude by ∼2.3 times. An Allan deviation of the QEPAS sensor performance based on the custom QTF was evaluated by tuning the laser wavelength away from the H_2_O absorption line. With an integration time of ∼ 84 s, a detection limit of 6.3 ppm was achieved for H_2_O, corresponding to a normalized noise equivalent absorption (NNEA) coefficient of 1.7 × 10^−8^ cm^-1^∙W∙Hz^-1/2^ in the case of a bare QTF without acoustic resonators. The achieved NNEA is 10 times better than that of a commercial standard QTFs. Such custom QTF with a 12.5 % lower resonance frequency and a smaller prong spacing benefit the photoacoustic detection of molecules with a low V-T relaxation rate such as CO_2_ and NO_2_. The performance of the custom tuning fork can be further enhanced by use of acoustic resonators in on-beam or off-beam configuration. The on-beam configuration can provide a ∼30 times enhancement in sensitivity by the strong coupling effect between the QTF and the two resonator tubes [43]. The off-beam configuration will benefit the using of laser sources with poor beam quality such quantum cascade lasers and light-emitting diodes [44]. In this work, benefiting from the performance of the custom QTF, only a bare QTF was employed as the QEPAS spectrophone, simplify the sensor structure and enhance the robustness. The pilot line manufactured custom QTFs with lower resonance frequencies and higher *Q* factors shows the opportunity on the mass production of QEPAS instruments based on custom QTFs. Next step is to develop cost-effective custom tuning forks with a resonance frequency ∼ 10 kHz or less by pilot lines. Not only for quartz-enhanced photoacoustic spectroscopy, another important application of the developed QTF is the atomic force microscope (AFM) in which the uniformity on resonance frequencies is of significant importance [38]. Considering the piezoelectric effect in quartz crystal is not so strong, further improvement can be made by using of traditional piezoelectric materials such as lead zirconate titanate (PZT), barium titanate (BaTiO_3_). The custom tuning fork with piezoelectric coefficients hundreds of times higher than the quartz can be expected to achieve the unprecedented gas detection limit.

## Funding

This work is supported by the National Natural Science Foundation of China (61675092, 61601404, 61705086), Natural Science Foundation of Guangdong Province (2016A030313079, 2016A030310098, 2016A030311019, 2019A1515011380), Special Funds for Major Science and Technology Projects of Guangdong Province (2019B010138004, 2015B010125007), Project of Guangzhou Industry Leading Talents (CXLJTD-201607), and Planned Science & Technology Project of Guangzhou (2017A010102006, 2016A010101017, 2016B010111003, 201506010046), Joint fund of pre-research for equipment, Ministry of Education of China (6141A02022124), Aeronautical Science Foundation of China (201708W4001); Foundation for Distinguished Young Talents in Higher Education of Guangdong (2018KQNCX009, 2018KQNCX279), the Fundamental Research Funds for the Central Universities (21619402), State Key Laboratory of Applied Optics (SKLAO-201914). Frank Tittel acknowledges the financial support from the US National Science Foundation (NSF) ERC MIRTHE award, a NSF NeTS Large “ASTRO” award (No. R3H685) and a grant C-0586 from the Welch Foundation.

## Declaration of Competing Interest

The authors declare that there are no conflicts of interest.
